# Simple Rapid Production of Calcium Acetate Lactate from Scallop Shell Waste for Agricultural Application

**DOI:** 10.3390/ijms26104488

**Published:** 2025-05-08

**Authors:** Sorakit Mongkol, Somkiat Seesanong, Banjong Boonchom, Nongnuch Laohavisuti, Wimonmat Boonmee, Somphob Thompho, Pesak Rungrojchaipon

**Affiliations:** 1 Department of Chemistry, King Mongkut’s Institute of Technology Ladkrabang, Bangkok 10520, Thailand; sorakit.mo@kmitl.ac.th (S.M.); pesak.ru@kmitl.ac.th (P.R.); 2 Office of Administrative Interdisciplinary Program on Agricultural Technology, King Mongkut’s Institute of Technology Ladkrabang, Bangkok 10520, Thailand; somkiat.se@kmitl.ac.th; 3 Material Science for Environmental Sustainability Research Unit, King Mongkut’s Institute of Technology Ladkrabang, Bangkok 10520, Thailand; 4 Municipal Waste and Wastewater Management Learning Center, King Mongkut’s Institute of Technology Ladkrabang, Bangkok 10520, Thailand; 5 Department of Animal Production Technology and Fishery, School of Agricultural Technology, King Mongkut’s Institute of Technology Ladkrabang, Bangkok 10520, Thailand; 6 Department of Biology, King Mongkut’s Institute of Technology Ladkrabang, Bangkok 10520, Thailand; wimonmat.bo@kmitl.ac.th; 7 Faculty of Pharmaceutical Sciences, Chulalongkorn University, 254 Phayathai Road, Patumwan, Bangkok 10330, Thailand; somphob.t@chula.ac.th

**Keywords:** calcium acetate lactate, scallop shell waste, renewable calcium source, thermal decomposition, multi-anionic calcium compound

## Abstract

Calcium acetate lactate (CAL) was rapidly synthesized for the first time using the reaction between the scallop shell-derived calcium carbonate (CaCO_3_) and the binary phase of acetic and lactic acids. Calcium acetate (CA) and calcium lactate (CL) synthesized from the reaction of scallop shell-derived CaCO_3_ with each acid by similarity routes are compared with the obtained CAL product. The production yields are 88.24, 79.17, and 96.44%, whereas the solubilities are 93.77, 90.18, and 95.08% for CA, CL, and CAL, respectively. All the synthesized CA, CL, and CAL samples were characterized and confirmed by X-ray fluorescence (XRF) to examine the calcium main element and other impurities of minor elements, X-ray diffraction (XRD) to investigate the crystallography, Fourier transform infrared (FTIR) to characterize the vibrational characteristics of the functional groups, scanning electron microscope (SEM) to observe the sample morphologies, and the thermogravimetric analysis (TGA) to investigate the thermal decomposition processes of samples. The experimental results pointed out that the synthesized CA, CL, and CAL were the monohydrate, pentahydrate, and dihydrate forms with chemical formulae of Ca(CH_3_COO)_2_·H_2_O, Ca(CH_3_CHOHCOO)_2_·5H_2_O, and Ca(CH_3_COO)(CH_3_CHOHCOO)·2H_2_O, respectively. The final thermal decomposition product of all calcium compounds was calcium oxide (CaO). The CAL sample’s vibrational characteristics, crystal phases, and morphologies show the binary acetate and lactate anion phases, confirming the new binary anionic calcium acetate lactate obtained. In conclusion, this research proposes an easy and low-cost technique to prepare a new valuable CAL compound using scallop shell waste as a cheap and renewable calcium source.

## 1. Introduction

Many marine shell manufacturers along the east and south of Thailand (a country in Southeast Asia) have produced a large number of shell wastes, and most of them are dumped into landfills [1]. Moreover, enormous quantities of shell waste, especially scallops, oysters, mussels, and cockles, have affected the high cost of waste treatment. This environmental problem has accumulated over many years, which motivates researchers to find superior methods to solve this problem, and recycling is the green method to treat shell waste. Many researchers have tried to find an alternative calcium source to reduce the production cost of various advanced calcium compounds, and waste is an excellent choice for a renewable calcium source to solve problems and receive the benefits associated with this waste. Scallop waste, one of the most abundant shell wastes, contains a very high percentage of calcium carbonate (CaCO_3_), more than 98% [2]; it was therefore considered and used as a renewable calcium source to prepare bio-materials such as calcium oxide (CaO), calcium hydroxide (Ca(OH)_2_), calcium acetate (Ca(CH_3_COO)_2_), calcium lactate (Ca(CH_3_CHOHCOO)_2_), etc. [1,2,3,4].

Calcium acetate (CA) and calcium lactate (CL), important organic calcium compounds, is composed of a calcium cation (Ca^2+^) and two anions (acetate, CH_3_COO^−^ and lactate, CH_3_CHOHCOO^−^). Both compounds can be used in the chemical industry as a starting reactant to produce valuable compounds such as nano CaO for further application as the sorbent to capture greenhouse gases (carbon dioxide, CO_2_) [5]. CA can also be used in the environmental field as a powerful agent to control the emission of sulfur dioxide (SO_2_), nitrogen oxide (NO_x_), and other toxic gasses in coal combustion processes [6]. In addition, CA can be used in the medical field to treat hyperphosphatemia (too much phosphate in the blood) symptoms [7]. According to previous research, CA could be synthesized from the reaction between acetic acid (CH_3_COOH) and CaCO_3_. Thongkam et al. used scallop shell-derived CaCO_3_ as a raw material to prepare CA [3]. Each concentration of four acetic acid concentrations (40, 50, 60, and 70 wt%) was reacted to the CaCO_3_ at ambient temperature until the completely dried powder was obtained. The physicochemical properties of the synthesized CAs were then investigated. When the acetic acid at a concentration of 60 wt% was used in the preparation process, a maximum CA yield of 87% was obtained with a short drying time at a low temperature [3]. In comparison, CL has been applied as a material to form an efficient bio-concrete with improved compressive strength [8,9,10,11,12], as a coagulant for removing suspended solids from water [13], and as a calcium source for animals and plants [13]. CL has been used to keep and extend the shelf-life of flowers, fruits, and vegetables [13]. It has also been used as a food additive with E number E327, classified by the United States Food and Drug Administration (U.S. FDA) as Generally Recognized as Safe (GRAS) [14], as a stabilizer and thickener, nutritional supplement, leavening agent, flavor enhancer or flavoring agent, and firming agent [15], as a calcium source for preventing and treating calcium deficiencies [10], as an antidote for soluble fluoride ingestion [16] and as a calcium source for hypocalcemia (calcium deficiency) symptoms [17] and for the prevention of tetany, an anti-tartar agent in some mouthwashes and toothpastes, and as an antacid [18]. The importance of using both organic calcium compounds has led to our interest in synthesizing compounds containing both anions in the structure of a calcium compound. Calcium acetate lactate (Ca(CH_3_COO)(CH_3_CHOHCOO)_2_), new calcium binary organic anions, was not reported anywhere and may be used for many applications to replace each singly calcium organic compound. In particular, agricultural applications such as organic calcium liquid fertilizer and post-harvest shelf-life extenders for vegetables and fruits.

Binary or multi-anionic calcium compounds such as calcium acetate chloride pentahydrate (Ca(CH_3_COO)Cl·5H_2_O or calclacite mineral) and tricalcium triacetate chloride dinitrate hexahydrate (Ca_3_(CH_3_COO)_3_Cl(NO_3_)_2_·6H_2_O or thecotrichite mineral) were found in the natural materials such as limestone rocks and fossils [19]. These minerals were also observed in ceramics [20], pottery shards [21], and ancient reliefs [22]. The occurrence of an efflorescence salt (grey or white crystalline salts on the exterior or interior of the shells) such as calcium acetate formate monohydrate (Ca(CH_3_COO)(HCOO)·H_2_O) on mollusk shells was reported by Tennent and Baird [23]. A complex efflorescence salt, such as dicalcium acetate formate dinitrate tetrahydrate (Ca_2_(CH_3_COO)(HCOO)(NO_3_)_2_·4H_2_O) was investigated and reported by Bette et al. [24]. These white efflorescent crystals were observed on the surfaces of a bronze bowl, classical ceramic, and ancient wine jugs 24]. Most of the efflorescence salts are poorly characterized as they usually occur as multiphase samples. The formation of dicalcium triacetate nitrate dihydrate (Ca_2_(CH_3_COO)_3_(NO_3_)·2H_2_O) [25] and the dehydration-rehydration behavior of calclacite (Ca(CH_3_COO)Cl·5H_2_O) [26] were investigated and reported. Another efflorescence salt, such as heptacalcium hexaacetate octaformate pentahydrate (Ca_7_(CH_3_COO)_6_(HCOO)_8_·5H_2_O) was also investigated [23]. The crystal structures of some multi-anionic calcium compounds such as Ca(CH_3_COO)Cl·5H_2_O (calclacite) [27], Ca_2_(CH_3_COO)_3_(NO_3_)·2H_2_O [25], Ca_3_(CH_3_COO)_3_Cl(NO_3_)_2_·6H_2_O (thecotrichite) [28], and Ca_2_(CH_3_COO)(HCOO)(NO_3_)_2_·4H_2_O [24] were investigated using an XRD technique. Bette et al. synthesized two calcium acetate formate forms, namely Ca_3_(CH_3_COO)_4_(HCOO)_2_·4H_2_O and Ca(CH_3_COO)(HCOO)·H_2_O, at room temperature using the ternary system, comprising calcium acetate, calcium formate, and water (Ca(CH_3_COO)_2_-Ca(HCOO)_2_·H_2_O) [29]. They used elemental and thermal analyses to determine the phase compositions and used Raman and infrared (IR) spectroscopies to confirm the presence of formate (HCOO^−^) and acetate (CH_3_COO^−^) anions in both synthesized forms.

So far, some binary and ternary-anionic calcium compounds have been discovered and synthesized, as mentioned above. However, a di-anionic calcium compound such as calcium acetate lactate (CAL, Ca(CH_3_COO)-Ca(CH_3_CHOHCOO)) was not synthesized and investigated. Consequently, this research aims to synthesize CA, CL, and CAL using natural shell waste as an alternative and renewable calcium source. Using scallop shells as a renewable calcium source not only reduces environmental problems but also increases the value-added scallop shell waste. The waste was cleaned and ground first to obtain the scallop-shell-derived CaCO_3_ powder. This CaCO_3_ powder was then reacted with acetic acid (CH_3_COOH), lactic acid (CH_3_CHOHCOOH), or a mixture of these acids, causing the formation of CA, CL, and CAL, respectively. An X-ray fluorescence (XRF) analyzer was applied to characterize and confirm the chemical composition of the synthesized calcium compounds. X-ray diffraction (XRD) was performed to investigate sample crystallography. The vibrational characteristics of the functional groups in the synthesized compounds were investigated using a Fourier transform infrared (FTIR) spectrophotometer and were assigned according to acetate and lactate anions. The morphologies of the samples were observed by scanning electron microscope (SEM). Finally, thermogravimetric analysis (TGA) was also used to investigate the thermal decomposition behaviors of samples through the thermogravimetric (TG) and differential thermogravimetric (DTG) techniques.

## 2. Experiments

### 2.1. Materials

Scallop shell waste was collected from Chonburi (Eastern), Thailand seafood restaurants. First, the waste was washed with distilled water to remove dust and some shells’ tissue, then washed with sodium hypochlorite (14% NaOCl, Merck/Burlington, MA, USA) to remove residual shells’ tissue and other organic compounds from the wastes [5]. The waste was finally washed with distilled water to ensure the complete washing process. The cleaned waste was dried in an oven (105 °C, 3 h), ground using a mechanical process, and subsequently sieved through 50 mesh to obtain white CaCO_3_ powder. Two chemicals, namely acetic acid (99.7 wt%, 17.416 mol/L CH_3_COOH, Merck/Burlington, MA, USA) and lactic acid (88 wt%, 13.310 mol/L C_2_H_4_OHCOOH, Merck/Burlington, MA, USA), of commercial grade, were also used as starting materials without further purification. A 10 mol/L acetic acid solution was prepared using deionized water as a solvent. At the same time, a 10 mol/L lactic acid solution was simultaneously prepared.

### 2.2. Synthesis

The synthesis of CA and CL samples was conducted using an exothermic reaction. To prepare CA or CL, the shell-derived CaCO_3_ powders were mixed with 10 mol/L acetic acid or 10 mol/L lactic acid at a mole ratio of CaCO_3_:acid of 1:2. The obtained mixture was stirred continuously (50 rpm) with the elimination of CO_2_ gas. The stirring process was finished when CO_2_ was evaporated completely, and the completion time was about 30 min. After that, the resulting mixture was exposed to the ambient air conditions to obtain dried powders. The dried products were ground and sieved through 50 mesh, and the CA or CL powders were obtained. The powder products, synthesized from the reactions that used 10 mol/L acetic acid and 10 mol/L lactic acid, were labeled as CA and CL, respectively. Calcium acetate lactate (CAL) was also prepared by a process similar to the abovementioned, but an acetic acid solution (10 mol/L) was first mixed with the lactic acid solution (10 mol/L). A beaker containing the shell-derived CaCO_3_ powders was slowly added by the mixture between acetic acid and lactic acid in the mole ratio of the CaCO_3_ powder to acetic acid to lactic acid of 1:1:1. When exposing the reaction mixture to the air, the mixture was dried, and the dried product was then ground, sieved, and labeled as CAL.

### 2.3. Characterizations

The chemical compositions of all synthesized calcium compounds (CA, CL, and CAL) were analyzed by a SRS 3400 X-ray fluorescence (XRF) spectrophotometer (XRF, SRS 3400, Bruker, Billerica, MA, USA). To prepare the XRF sample without contamination, agate mortar was used to pulverize and homogenize a sample. The homogenized sample was then pressed into a pellet using starch as a binder before XRF measurement [30]. The MiniFlex X-ray diffractometer (Bruker AXS, Billerica, MA, USA) was used to investigate the crystallography of samples. The crystallography investigation was conducted under ambient conditions using a 2 theta (2θ) range of 5–60°, an increment of 0.02°, and a scan speed of 1 s/step [31]. The experimental XRD patterns of samples were compared with the Powder Diffraction File (PDF) database of the International Center for Diffraction Data (ICDD). The vibrational spectroscopy of samples was analyzed by using a Spectrum GX Fourier transform infrared (FTIR) spectrophotometer (Spectrum GX, PerkinElmer, Waltham, MA, USA). Each sample was mixed and homogenized with potassium bromide (KBr, spectroscopic grade). A manual hydraulic press was applied to press the mixture at 2 tons for 1 min, forming a sample pellet. The infrared spectrum of each pellet was recorded from 4000 to 400 cm^−1^ with a resolution of 2 cm^−1^ using a 30-scan number to increase the signal [31]. A 1450 VP scanning electron microscope (SEM, VP1450, LEO, North Billerica, MA, USA) was used to analyze sample morphologies. An alumina stub was adhered to each sample using double-sided conductive tape, and the sample was then coated with gold powder using the sputtering technique before the SEM process [32]. The thermal decomposition behavior of samples was investigated by the thermogravimetric analytic (TGA) technique on a Pyris Diamond TG/DTA instrument (TG/DTA Pyris Diamond, PerkinElmer, Waltham, MA, USA). Each sample and calcined α-Al_2_O_3_ were placed into the sample and reference TG pans, respectively. The thermal decomposition was conducted under an N_2_ flow rate of 100 mL/min from 30 to 900 °C at a heating rate of 10 °C/min [33]. The solubility of products was investigated by the following: Typical process: 10 g of each sample was dissolved in 100 mL DI water and then continuously stirred at 100 rpm at room temperature for 1 h. The insoluble fraction (solid) was separated by filtration with a suction pump and dried in an oven (100 °C for 1 h) to determine the weight of the dried solid, which was used to estimate the % solubility. Triplicate experiments were performed for each sample.

## 3. Results and Discussion

Equations (1)–(3) show the chemical reactions between the scallop-shell-derived CaCO_3_ powder and acetic acid (CH_3_COOH), lactic acid (CH_3_CHOHCOOH), or a mixture of these acids, resulting in the formation of hydrate forms of calcium acetate (Ca(CH_3_COO)_2_·*x*H_2_O), calcium lactate (Ca(CH_3_CHOHCOO)_2_·*x*H_2_O), and calcium acetate lactate (Ca(CH_3_COO)(CH_3_CHOHCOO)·*x*H_2_O) products, respectively.

Calcium acetate (CA)  CaCO_3_(s) + 2CH_3_COOH(aq) → Ca(CH_3_COO)_2_·*x*H_2_O(s) + CO_2_(g) (1)

Calcium lactate (CL)  CaCO_3_(s) + 2CH_3_CHOHCOOH(aq) → Ca(CH_3_CHOHCOO)_2_·*x*H_2_O(s) + CO_2_(g) (2)

Calcium acetate lactate (CAL)  CaCO_3_(s) + CH_3_COOH(aq) + CH_3_CHOHCOOH(aq) → Ca(CH_3_COO)(CH_3_CHOHCOO)·*x*H_2_O(s) + CO_2_(g) (3)

From the preparation according to Equations (1)–(3), the parameters investigated, including production yield, reaction time, and soluble fraction, of all prepared samples were obtained, and the results are reported in Table 1. It was found that the reaction time of CAL is between that of CA and CL. The higher yield and solubility were observed for the CAL sample, while lower values for both parameters were detected for the CL sample.

### 3.1. Chemical Composition

An XRF spectrophotometer was used to examine the chemical compositions of all the synthesized calcium compounds, and the results are listed in Table 2. The experimental results exhibit that all synthesized calcium samples consisted mostly of CaO at higher than 96.5 wt% without element toxicity. However, other trace chemical compositions were also observed in oxide forms, but they are useful to apply in agriculture.

According to the results, when acetic acid or lactic acid with the selected concentrations were used in the preparation process, all resulting products, mono-anionic calcium compounds (CA and CL), showed high CaO contents with low amounts of other chemical compositions. A similar result was obtained in the case of binary anionic calcium compound (CAL). It can therefore be concluded that all the concentrations of acetic acid and/or lactic acid could be successfully employed to prepare calcium acetate (Ca(CH_3_COO)_2_), calcium lactate (Ca(CH_3_CHOHCOO)_2_), and calcium acetate lactate (Ca(CH_3_COO)(CH_3_CHOHCOO)) compounds.

### 3.2. Vibrational Spectroscopy

**Figure 1:**
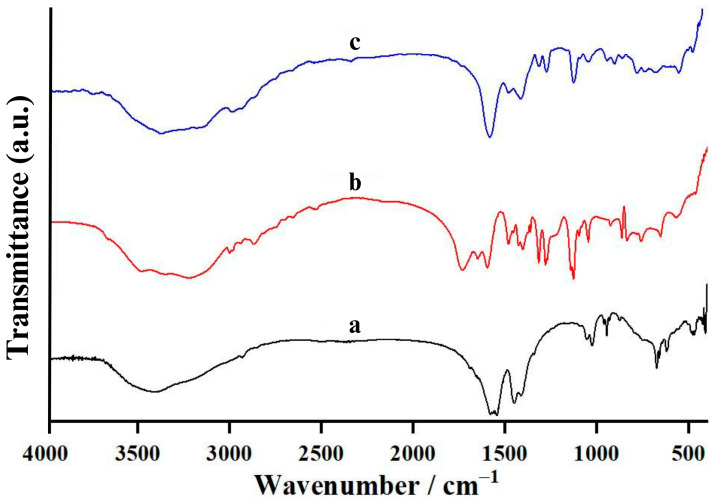
Fourier transform infrared (FTIR) spectra of synthesized (**a**) calcium acetate (CA), (**b**) calcium lactate (CL), and (**c**) calcium acetate lactate (CAL).

An FTIR spectrophotometer was employed to characterize the vibrational characteristics of the functional groups (i.e., acetate (CH_3_COO^−^) and lactate (CHOHCOO^−^) anions, water (H_2_O), and calcium oxide (Ca-O)) in all synthesized calcium compounds. The vibrational characteristics of all samples in the wavenumber range of 4000–400 cm^−1^ were then clarified. Figure 1 shows the infrared spectra of CA, CL, and CAL synthesized from 10 mol/L acetic acid, lactic acid, and binary acid phase and scallop-derived CaCO_3_. All vibrational characteristics (Figure 1a) pointed out that the reaction between scallop-derived CaCO_3_ and CH_3_COOH could form a calcium acetate hydrate compound with the chemical formula of Ca(CH_3_COO)_2_·*x*H_2_O because of the presence of the vibrational characteristics of acetate (CH_3_COO^–^) anion, water (H_2_O), and calcium oxide (Ca–O) functional groups. The corresponding vibrational modes and vibrational positions (wavenumber) are presented in Table 3. However, the mole number of water (*x* value) will be investigated and described in the thermal decomposition section. The vibrational characteristics of Ca(CH_3_COO)_2_·*x*H_2_O observed in this work are in good agreement with the vibrational spectroscopic results reported by Musumeci et al. for Ca(CH_3_COO)_2_·H_2_O (calcium acetate monohydrate) and Ca(CH_3_COO)_2_·0.5H_2_O (calcium acetate hemihydrate) [34], by Bette et al. for Ca(CH_3_COO)_2_·H_2_O and Ca(CH_3_COO)_2_ (anhydrous calcium acetate) [35], and by Koleva for CaH(CH_3_COO)_3_·H_2_O (calcium hydrogen triacetate monohydrate) [36].

Figure 1b demonstrates the vibrational spectra of CL products (Ca(CH_3_CHOHCOO)_2_). The vibrational positions and their vibrational modes are presented in Table 3. All CL samples showed similar vibrational characteristics, confirming the presence of the same functional group, namely, lactate (CH_3_CHOHCOO^−^), H_2_O, and Ca–O. All vibrational characteristics are listed in Table 4 and confirm that the reacted product from the reaction between scallop-derived CaCO_3_ and CH_3_CHOHCOOH is calcium lactate hydrate with the chemical formula of Ca(CH_3_CHOHCOO)_2_·*x*H_2_O. The vibrational characteristics obtained in this work are in good agreement with the information reviewed and reported by Pavia et al. for the spectroscopies of various functional groups (including lactate anion) [37], by Cassanas et al. for lactic acid and lactate anion [38], and by Cheong for Ca(CH_3_CHOHCOO)_2_·5H_2_O (calcium lactate pentahydrate) synthesized by aragonite-phase CaCO_3_ [39]. Moreover, Lee and Kim synthesized CL from black-snail-derived CaCO_3_, and infrared spectroscopy was then employed to investigate the vibrational spectroscopy of the CL [40], which was in good agreement with this work.

The infrared spectrum of binary anionic calcium compound (calcium acetate lactate (CAL), Ca(CH_3_COO)(CH_3_CHOHCOO) was presented in Figure 1c. Table 5 presents the vibrational positions and their vibrational modes, which indicate the vibrational characteristics of acetate (CH_3_COO^–^), lactate (CH_3_CHOHCOO^−^), H_2_O, and Ca–O functional groups. The presence of acetate and lactate vibrational characteristics, listed in Table 5, confirmed that calcium acetate lactate hydrate with the chemical formula of Ca(CH_3_COO)(CH_3_CHOHCOO)·*x*H_2_O was formed from the reaction of scallop-derived CaCO_3_, CH_3_COOH, and CH_3_CHOHCOOH. In comparison, the *x* value (mole number of water) could be determined by the thermal decomposition technique.

### 3.3. Thermal Decomposition

**Figure 2:**
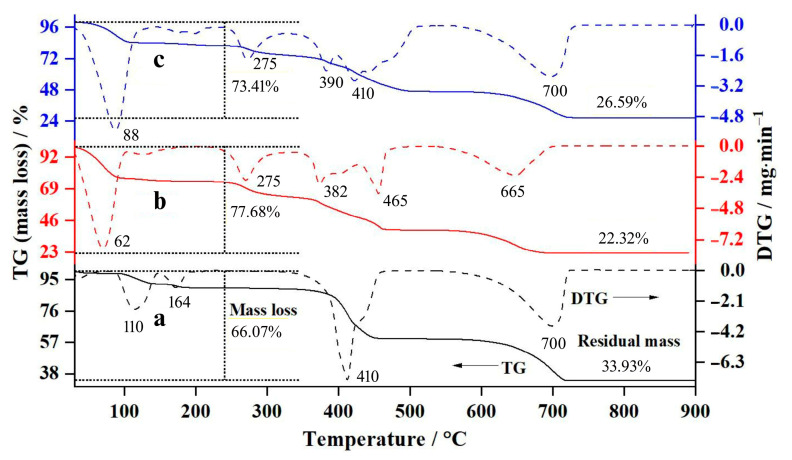
Thermal decomposition (TG and DTG) curves of the synthesized (**a**) calcium acetate (CA), (**b**) calcium lactate (CL6), and (**c**) calcium acetate lactate (CAL).

The thermal decomposition processes of all the synthesized calcium compounds were investigated by the TG/DTA technique, and the obtained thermal behaviors were then analyzed. Figure 2 shows the TG and DTG curves of CA and CL in the temperature range of 30–900 °C. The change in the mass loss (%), as shown in the TG curves, related well to their differential patterns (DTG peaks). The thermal characteristics of the calcium acetate (CA) or calcium lactate (CL) groups are similar to each other within the group. Therefore, it could be concluded that the selected ranges of acetic or lactic acid concentrations can be employed successfully for the synthesis of CA or CL.

The thermal decomposition of CA, as shown in Figure 2a, was also described. The first step of the mass loss (30–200 °C) was assigned as the elimination of the crystalline water of calcium acetate hydrate, and this water elimination was called the “dehydration process”, resulting in the formation of an anhydrous phase (Ca(CH_3_COO)_2_) [35]. The obtained mass-loss percentages of 9.63% agreed with the theoretical value (10.23%), confirming the dehydration process. The second step (200–470 °C), with a mass-loss percentage of 29.98%, was assigned as the decomposition of Ca(CH_3_COO)_2_, forming the CaCO_3_ and acetone (CH_3_COCH_3_) products [5]. This thermal decomposition was called the “deacetonation process”. Furthermore, the thermal generated acetone further decomposed simultaneously to methane (CH_4_) and ketene (H_2_CCO) [40]. The final step (470–720 °C) with a mass-loss percentage of 25.92% was assigned as the decomposition of CaCO_3_, resulting in the formation of CaO and CO_2_, and this thermal decomposition was called the “decarbonization process” [5]. Therefore, the overall mass loss of the CA sample was 66.16%, whereas the residual mass was 33.84%.

Table 6 shows the experimental and theoretical values of the mass-loss and residual-mass percentages of calcium acetate (CA), confirming that the chemical formula of the synthesized CA was the Ca(CH_3_COO)_2_·H_2_O (calcium acetate monohydrate). The thermal decomposition of various solid-state compounds is a complex process; therefore, some experimental values of the mass-loss percentages in the thermal decomposition steps deviated slightly from the theoretical values [31,41]. However, the experimental values of the overall mass loss and residual mass percentages of the synthesized CA corresponded well with the theoretical values. Consequently, the thermal decomposition process of CA could be classified as three steps as demonstrated in Equations (4)–(6):

Dehydration process (30–200 °C) Ca(CH_3_COO)_2_·H_2_O(s) → Ca(CH_3_COO)_2_(s) + H_2_O(g) (4)

Deacetonation process (200–470 °C) Ca(CH_3_COO)_2_ (s) → CaCO_3_(s) + CH_3_COCH_3_(g) (5)

Decarbonization process (470–720 °C) CaCO_3_(s) → CaO(s) + CO_2_(g) (6)

Figure 2b displays the thermal decomposition behaviors of the synthesized CL compound. The TG curves for all CL presented three mass-loss steps, appearing in the ranges of 30–170, 170–480, and 480–690 °C, respectively. Three decomposition steps corresponded to (*i*) the dehydration process of calcium lactate hydrate to form an anhydrous phase (Ca(CH_3_CHOHCOO)_2_), (*ii*) the elimination of ethyl lactate (C_5_H_10_O_3_ or CH_3_CHOHCOOC_2_H_5_) to form CaCO_3_, and (*iii*) the decarbonation process of CaCO_3_ to form CaO, respectively. The experimental and theoretical values of the mass-loss and residual-mass percentages of calcium lactate (CL) are listed in Table 6, confirming that the chemical formula of the synthesized CL was Ca(CH_3_CHOHCOO)_2_·5H_2_O (calcium lactate pentahydrate). Consequently, the thermal decomposition mechanism of CL can be written as (Equations (6)–(8)):

Dehydration process (30–170 °C) Ca(CH_3_CHOHCOO)_2_·5H_2_O(s) → Ca(CH_3_CHOHCOO)_2_(s) + 5H_2_O(g) (7)

Elimination of ethyl lactate (170–480 °C) Ca(CH_3_CHOHCOO)_2_(s) → CaCO_3_(s) + CH_3_CHOHCOOC_2_H_5_(g) (8)

Decarbonization process (480–690 °C) is the same reaction in Equation (6).

The thermal decomposition behavior of the synthesized CAL was also studied, and its TG and DTG curves are displayed in Figure 2c. To compare the thermal decomposition behavior of CAL, the TG and DTG curves of CA and CL are also included in Figure 2a,b. Three thermal decomposition steps of the CAL, which appeared in the range of 30–130, 130–510, and 510–730 °C, corresponded to (*i*) the dehydration process of calcium acetate lactate hydrate to form anhydrous phase (Ca(CH_3_COO)(CH_3_CHOHCOO)), (*ii*) the elimination of acetoin (CH_3_CHOHCOCH_3_) to form CaCO_3_, and (*iii*) the decarbonation process of CaCO_3_ to form CaO, respectively. The values between the experimental and theoretical mass-loss and residual-mass percentages of CAL, as demonstrated in Table 5, indicated that the chemical formula of the synthesized CAL was the Ca(CH_3_COO)(CH_3_CHOHCOO)·2H_2_O (calcium acetate lactate dihydrate). The thermal behavior of CAL is similarly obtained by the combination of the thermal behavior of CA and CL, which indicates the obtained binary organic form. Consequently, the thermal decomposition mechanism of CAL can be written as (Equations (6), (9), and (10)):

Dehydration process (30–130 °C) Ca(CH_3_COO)(CH_3_CHOHCOO)·2H_2_O(s) → Ca(CH_3_COO)(CH_3_CHOHCOO)(s) + 2H_2_O(g) (9)

Elimination of acetoin (130–510 °C) Ca(CH_3_COO)(CH_3_CHOHCOO)(s) → CaCO_3_(s) + CH_3_CHOHCOCH_3_(g) (10)

Decarbonization process (510–730 °C) is the same reaction in Equation (6).

### 3.4. Crystallography

**Figure 3:**
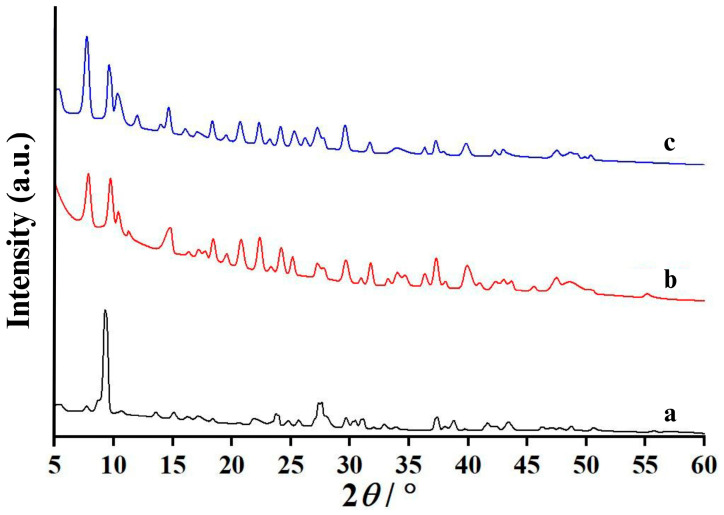
X-ray diffraction (XRD) patterns of the synthesized (**a**) calcium acetate (CA), (**b**) calcium lactate (CL), and (**c**) calcium acetate lactate (CAL).

An X-ray diffractometer was used to investigate the crystallography of all the synthesized calcium compounds, and the resulting XRD patterns of CA and CL are displayed in Figure 3. The prepared CA sample, as demonstrated in Figure 3a, shows similar XRD patterns, pointing out the same crystal structure. After comparing to ICDD data of PDF # 010-0776, it could be concluded that CA was calcium acetate monohydrate (Ca(CH_3_COO)_2_·H_2_O) [34]. Klop et al. used the Patterson–Fourier method to investigate the crystal structure of Ca(CH_3_COO)_2_·H_2_O and have reported that the crystal structure of Ca(CH_3_COO)_2_·H_2_O composed of infinite multiple O-bridged double-stranded Ca chains, which were cross-linked by the H bonds [42]. Ca(CH_3_COO)_2_·H_2_O crystallizes in triclinic crystal system with the lattice parameters *a* = 6.751 Å, *b* = 11.077 Å, *c* = 11.783 Å, the lattice angles *α* = 116.50°, *β* = 92.41°, *γ* = 97.32°, the unit cell volume of 777.1 Å^3^, and a *Z* number of 4 (number of formula units in the unit cell). The diffraction pattern of CA obtained in this work is in good agreement with data reported by Musumeci et al. [34], demonstrating that 10 mol/L acetic acid was successfully employed to prepare CA with the chemical formula of Ca(CH_3_COO)_2_·H_2_O.

Figure 3b shows the diffraction pattern of the CL product prepared using 10 mol/L lactic acid with the scallop-shell-derived CaCO_3_ powder. The diffraction patterns of CL compounds were determined and reported in a previous work. Mititelu et al. used Black Sea mussel shells as the precursor to synthesize CL, and according to ICDD data (PDF #029-1596), they concluded that the synthesized product was calcium lactate pentahydrate with the chemical formula of Ca(CH_3_CHOHCOO)_2_·5H_2_O [10]. Furthermore, the experimental results reported by Tansman et al. [9] were considered and used as a reference in this research study. Two forms of CL crystals, namely calcium L-lactate pentahydrate (L-CL) and calcium D-lactate pentahydrate (D-CL), were investigated and first reported by Johnson et al. [43]. D and L are Dexter and Laevus, which are the Latin words that mean right and left, respectively. The name “D or L” depends on the enantiomeric form of lactate anion (CH_3_CHOHCOO^−^) within the crystal form of CL [9]. “D-CL” means that CL contains two lactate groups in the right-handed enantiomeric form. In contrast, when two-lactate groups in the left-handed enantiomeric form are contained in a CL crystal, this CL is called “L-CL”. In addition, DL-CL crystals containing one D-lactate and one L-lactate have also been reported by Tansman et al. [9] and Johnson et al. [43]. Therefore, to correct these data, Tansman et al. [9] recommended that existing ICDD data of PDF # 029-1596 should be referred to for calcium DL-lactate pentahydrate or DL-CL only. The results reported by Cao et al. [44] suggested that L-CL is more soluble than DL-CL. Consequently, after comparing it to experimental data published by Tansman et al. [9], the synthesized CL in this research was confirmed as L-CL. As demonstrated in Figure 3b, the XRD pattern of CL is similar, confirming that 10 mol/L lactic acid was successfully employed to synthesize Ca(CH_3_CHOHCOO)_2_·5H_2_O in the L-form.

The XRD technique was also used to analyze CAL, and the results are presented in Figure 3c. This binary anionic calcium compound (calcium acetate lactate dihydrate, Ca(CH_3_COO)(CH_3_CHOHCOO)·2H_2_O) was synthesized for the first time; therefore, its experimental diffraction pattern observed in this work was not compared with either the ICDD database or other previous published research findings. However, when the diffraction pattern of Ca(CH_3_COO)(CH_3_CHOHCOO)·2H_2_O was compared with other synthesized calcium compounds, it was found that the diffraction pattern of Ca(CH_3_COO)(CH_3_CHOHCOO)·2H_2_O contained mixed patterns of CA and CL, but it is more similar to the diffraction pattern of calcium lactate pentahydrate (CL, Ca(CH_3_CHOHCOO)_2_·5H_2_O) than calcium acetate monohydrate (CA, Ca(CH_3_COO)_2_·H_2_O) as demonstrated in Figure 3a,b. Therefore, it could be concluded that the crystal structure of the synthesized Ca(CH_3_COO)(CH_3_CHOHCOO)·2H_2_O is similar to that of Ca(CH_3_CHOHCOO)_2_·5H_2_O.

### 3.5. Morphology

**Figure 4:**
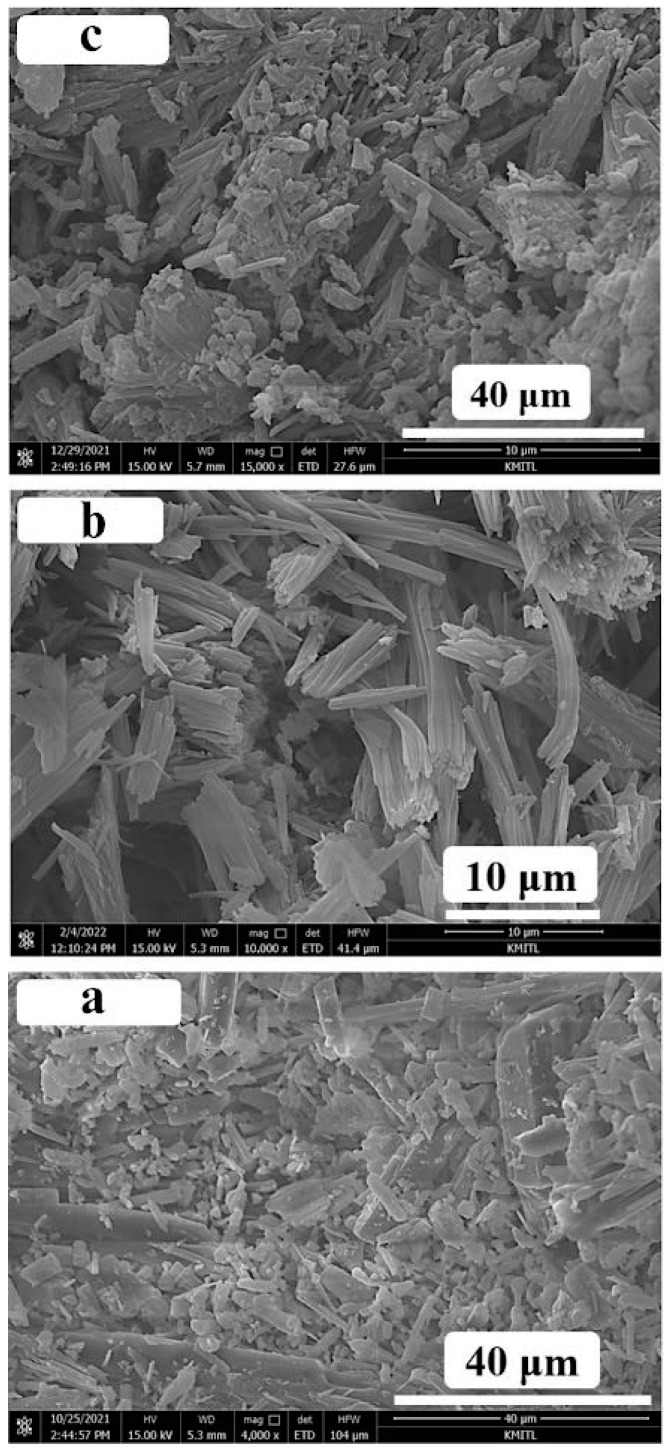
Scanning electron microscopic (SEM) images of the synthesized (**a**) calcium acetate (CA), (**b**) calcium lactate (CL), and (**c**) calcium acetate lactate (CAL).

The morphologies of all the calcium compounds derived from scallop-shell CaCO_3_ powders were observed by the SEM technique. The SEM image of the CA product is demonstrated in Figure 4a. The morphologies of the CA sample at the magnification of 4000 times show the plate-shaped crystals with different sizes from around 5 to 30 μm. The morphology of the CL compound at the magnification of 10,000 times is also demonstrated in Figure 4b, which shows large rod-shaped clusters with length and width ranges of around 2–20 and 0.2–1 µm, respectively. The SEM technique was also used to observe the morphology of the CAL product, and its SEM result at 15,000-time magnification is presented in Figure 4c. The morphology of CAL shows the same characteristics as the morphologies of both the CA and CL compounds. A mixture of plate/rod-shaped crystals with different sizes from around 5 to 35 μm was observed. Moreover, lots of small particles were assigned as the broken products of larger particles.

By converting discarded scallop shells into a potentially valuable new compound, this research contributes to the principles of circular economy and sustainable resource management. This approach not only reduces the environmental burden associated with shell disposal but also offers an alternative to conventional calcium sources, promoting a more environmentally friendly and economically viable route to produce calcium-based materials. The unique binary anionic structure of CAL warrants further investigation into its potential applications in various fields. For instance, its tailored solubility and thermal properties could be explored for applications in the food industry as a novel preservative or texture modifier or in the pharmaceutical sector as a calcium supplement with potentially enhanced bioavailability. Furthermore, the distinct morphology of CAL, exhibiting a combination of plate and rod-like features, suggests potential applications in material science, such as in the development of composite materials with specific mechanical or structural properties.

## 4. Conclusions

This work presents valuable information for preparing advanced calcium compounds using biowaste as a starting material. This useful scallop shell-derived CaCO_3_ material can be beneficially used to replace the usages of natural lime or dolomite ore obtained from non-living things, which are limited resources. After cleaning and grinding the shell waste, CaCO_3_ powder was obtained, which then was rapidly reacted with acetic acid or lactic acid or acetic-lactic acid to form CA, CL, and CAL, respectively. The XRF and XRD results confirm calcium to be the main element (97%) alongside other impurities of minor elements (3%) and the crystallography of the synthesized products. The SEM images showed the plate-shaped crystals for CA, rod-shaped particles for CL, and a mixture of plate/rod-shaped particles for CAL. Dehydration (1st) and decarbonization (3rd) processes have occurred in all samples, whereas the deacetonation steps and elimination of ethyl lactate (2nd) process have occurred for CA, CAL, and CL, and the final thermal decomposition products of CA, CL, and CAL were observed to be CaO. The FTIR spectroscopic results confirmed the vibrational characteristics of acetate and lactate anions, water, and metal oxide in the synthesized calcium products. Consequently, all characterization techniques exhibited and confirmed that calcium acetate lactate dihydrate (Ca(CH_3_COO)(CH_3_CHOHCOO)·2H_2_O) was successfully synthesized by using the scallop shell waste as a renewable calcium source. These compounds obtained in this work will be applied in agricultural applications such as organic calcium liquid fertilizer and post-harvest shelf-life extenders for vegetables and fruits in further work.

## Figures and Tables

**Table 1:** The production parameters of CA, CL, and CAL from scallop-shell-derived CaCO_3_ powder and different acids.

Samples	Reaction Time (min)	Production Yields (%)	Soluble Fractions (%)
CA	65	88.24 ± 1.26	93.77 ± 1.42
CL	2	79.17 ± 1.13	90.18 ± 1.36
CAL	26	96.44 ± 1.24	95.08 ± 1.21

**Table 2:** Chemical compositions and contents of the synthesized calcium acetate (CA), calcium lactate (CL), and calcium acetate lactate (CAL) samples.

Chemical Compositions	Chemical Content/wt%		
	CA	CL	CAL
Calcium oxide (CaO)	97.60	97.40	96.80
Sodium oxide (Na_2_O)	0.35	0.33	0.47
Magnesium oxide (MgO)	0.23	0.21	0.52
Aluminum oxide (Al_2_O_3_)	0.07	0.07	0.05
Silicon dioxide (SiO_2_)	0.22	0.20	0.16
Phosphorus pentoxide (P_2_O_5_)	0.06	0.09	0.16
Sulfur trioxide (SO_3_)	0.92	1.13	1.34
Chloride (Cl^−^)	–	0.01	–
Potassium oxide (K_2_O)	0.02	0.03	0.03
Manganese oxide (MnO)	–	–	–
Ferric oxide (Fe_2_O_3_)	0.04	0.06	0.04
Strontium oxide (SrO)	0.50	0.52	0.46
Total	99.97	100.04	100.02

**Table 3:** Vibrational characteristics (mode) and vibrational positions (wavenumber) of all the synthesized calcium acetate (CA).

Vibrational Modes	Vibrational Symbols	Wavenumber/cm^−1^
Asymmetric O–H stretching of H_2_O	*ν*_as_(O–H)	3678–3286
Symmetric O–H stretching of H_2_O	*ν*_s_(O–H)	3286–3069
Asymmetric C–H stretching of CH_3_ of CH_3_COO^−^	*ν*_as_(H_2_C–H)	2984–2886
Symmetric C–H stretching of CH_3_ of CH_3_COO^−^	*ν*_s_(H_2_C–H)	2886–2814
H–O–H bending of H_2_O, asymmetric C=O and symmetric C=O stretching of COO^−^ of CH_3_COO^−^	*δ*(H_2_O), *ν*_as_(C=O) and *ν*_s_(C=O)	1720–1563
Asymmetric C–O stretching of COO^−^ of CH_3_COO^−^	*ν*_as_(C–O)	1563–1487
Symmetric C–O stretching of COO^−^ of CH_3_COO^−^	*ν*_s_(C–O)	1487–1429
Asymmetric CH_3_ bending of CH_3_COO^−^	*δ*_as_(CH_3_)	1429–1353
Symmetric CH_3_ bending of CH_3_COO^−^	*δ*_s_(CH_3_)	1353–1272
Out-of-plane CH_3_ bending of CH_3_COO^−^	*ρ*_op_(CH_3_)	1077–1039
In-plane CH_3_ bending of CH_3_COO^−^	*ρ*_ip_(CH_3_)	1039–981
C–C stretching of C–CH_3_ of CH_3_COO^−^	ν(C–C)	981–917
Symmetric O=C–O bending (twisting and rocking) of COO^−^ of CH_3_COO^−^	*δ*_st_(O=C–O) and *δ*_sr_(O=C–O)	695–665
Out-of-plane O=C–O stretching of COO^−^ of CH_3_COO^−^	*ρ*_op_(O=C–O)	665–597
Ca–O stretching	*ν*(Ca–O)	498–445
In-plane COO^−^ bending (rocking) of CH_3_COO^−^	*r*(COO^−^)	445–400

**Table 4:** Vibrational characteristics (mode) and vibrational positions (wavenumber) of all the synthesized calcium lactate (CL).

Vibrational Modes	Vibrational Symbols	Wavenumber/cm^−1^
Asymmetric O–H stretching of H_2_O	*ν*_as_(O–H)	3695–3316
Symmetric O–H stretching of H_2_O	*ν*_s_(O–H)	3316–3024
Asymmetric C–H stretching of CH_3_ of CH_3_CHOHCOO^−^	*ν*_as_(H_2_C–H)	3024–2961
Symmetric C–H stretching of CH_3_ of CH_3_CHOHCOO^−^	*ν*_s_(H_2_C–H)	2961–2917
C–H stretching of CH of CH_3_CHOHCOO^−^	*ν*(C–H)	2917–2826
H–O–H bending of H_2_O, asymmetric C=O and symmetric C=O stretching of COO^−^ of CH_3_CHOHCOO^−^	*δ*(H_2_O), *ν*_as_(C=O) and *ν*_s_(C=O)	1805–1418
Asymmetric CH_3_ bending (twisting) of CH_3_CHOHCOO^−^	*δ*_as_(CH_3_)	1513–1450
Symmetric CH_3_ bending (twisting and rocking) of CH_3_CHOHCOO^−^	*δ*_s_(CH_3_) and *r*(CH_3_)	1373–924
Symmetric C–H bending of CH of CH_3_CHOHCOO^−^	*δ*(CH)	1337–1246
C–C stretching of C–CH_3_ of CH_3_CHOHCOO^−^	*ν*(C–CH_3_)	1068–1015
C–C stretching of C–COO^−^ of CH_3_CHOHCOO^−^	*ν*(C–COO^−^)	883–842
Out-of-plane COO^−^ bending (twisting) of CH_3_CHOHCOO^−^	*t*(COO^−^)	842–765
Symmetric C–C bending of C–COH of CH_3_CHOHCOO^−^	*δ*(C–COH)	765–610
Out-of-plane COO^−^ bending (wagging) of CH_3_CHOHCOO^−^	*w*(COO^−^)	610–483
Ca–O stretching	*ν*(Ca–O)	483–442
In-plane COO^−^ bending (rocking) of CH_3_CHOHCOO^−^	*r*(COO^−^)	442–400

**Table 5:** Vibrational characteristics (mode) and vibrational positions (wavenumber) of all the synthesized calcium acetate lactate (CAL).

Vibrational Modes	Vibrational Symbols	Wavenumber/cm^−1^
Asymmetric O–H stretching of H_2_O	*ν*_as_(O–H)	3694–3213
Symmetric O–H stretching of H_2_O	*ν*_s_(O–H)	3213–3026
Asymmetric C–H stretching of CH_3_ of CH_3_COO^−^ and CH_3_CHOHCOO^−^	*ν*_as_(H_2_C–H)	3026–2846
Symmetric C–H stretching of CH_3_ of CH_3_COO^−^ and CH_3_CHOHCOO^−^	*ν*_s_(H_2_C–H)	2876–2814
C–H stretching of CH of CH_3_COO^−^ and CH_3_CHOHCOO^−^	*ν*(C–H)	2814–2711
H–O–H bending of H_2_O, asymmetric C=O and symmetric C=O stretching of COO^−^ of CH_3_COO^−^ and CH_3_CHOHCOO^−^	*δ*(H_2_O), *ν*_as_(C=O) and *ν*_s_(C=O)	1763–1452
Asymmetric CH_3_ bending (twisting) of CH_3_COO^−^ and CH_3_CHOHCOO^−^	*δ*_as_(CH_3_)	1509–1452
Symmetric CH_3_ bending (twisting and rocking) of CH_3_COO^−^ and CH_3_CHOHCOO^−^	*δ*_s_(CH_3_) and *r*(CH_3_)	1373–924
Symmetric C–H bending of CH of CH_3_COO^−^ and CH_3_CHOHCOO^−^	*δ*(CH)	1339–1246
C–C stretching of C–CH_3_ of CH_3_COO^−^ and CH_3_CHOHCOO^−^	*ν*(C–CH_3_)	1153–1004
C–C stretching of C–COO^−^ of CH_3_COO^−^ and CH_3_CHOHCOO^−^	*ν*(C–COO^−^)	977–839
Out-of-plane COO^−^ bending (twisting) of CH_3_COO^−^ and CH_3_CHOHCOO^−^	*t*(COO^−^)	839–758
Symmetric C–C bending of C–COH of CH_3_CHOHCOO^−^	*δ*(C–COH)	758–638
Out-of-plane COO^−^ bending (wagging) of CH_3_COO^−^ and CH_3_CHOHCOO^−^	*w*(COO^−^)	638–456
Ca–O stretching	*ν*(Ca–O)	456–423
In-plane COO^−^ bending (rocking) of CH_3_COO^−^ and CH_3_CHOHCOO^−^	*r*(COO^−^)	456–400

**Table 6:** Experimental and theoretical percentages of mass loss and residual mass of calcium acetate (CA), calcium lactate (CL), and calcium acetate lactate (CAL).

Samples	Steps	Temperatures/°C	DTG Peak/°C	Mass Losses/%		Residual Masses/%	
				Experiment	Theory	Experiment	Theory
CA	1st	30–200	110	10.24	10.23	89.76	89.77
	1st–2nd	30–470	410	40.85	43.19	59.15	56.81
	1st–3rd	30–720	700	66.07	68.17	33.93	31.83
CL	1st	30–170	62	25.84	29.22	74.16	70.78
	1st–2nd	30–480	225, 382, 465	61.14	67.54	38.86	32.46
	1st–3rd	30–690	665	77.68	81.81	22.32	18.19
CAL	1st	30–130	88	15.94	16.14	84.06	83.89
	1st–2nd	30–510	275, 390, 410	53.52	55.61	46.48	44.39
	1st–3rd	30–730	700	73.41	75.33	26.59	24.67

## Data Availability

Data will be made available on request.
